# From surgery to rehabilitation: a cross-sectional survey on the feasibility of live digital ward rounds between acute care and rehabilitation clinics in trauma patients

**DOI:** 10.1007/s00068-026-03084-w

**Published:** 2026-03-17

**Authors:** Lina Schreiber, Sascha Halvachizadeh, Frank Peter Schäfer, Felix Tschui, Christian Sturzenegger, Sönke Johannes, Hans-Christoph Pape, Sandro Michael Heining

**Affiliations:** 1 Department of trauma, University Hospital Zurich, University of Zurich, Raemistrasse 100, 8091 Zurich, Switzerland; 2 Bellikon rehabilitation clinic, Mutschellenstrasse 2, 5454 Bellikon, Switzerland; 3 Zurzach Care rehabilitation clinic, Quellenstrasse 34, 5330 Bad Zurzach, Switzerland

**Keywords:** Telemedicine, Remote consultation, Acute care, Perioperative care, Rehabilitation medicine, Health personnel, Multiple trauma, Wounds and injuries

## Abstract

**Introduction:**

Telemedicine is increasingly embraced by both patients and healthcare professionals. By bridging the gap between acute care hospitals and rehabilitation clinics, it offers a promising approach to enhance communication and continuity of care. This study aimed to assess the feasibility of telemedicine in the rehabilitation of patients with musculoskeletal injuries or polytrauma.

**Methods:**

A cross-sectional survey was conducted involving healthcare professionals engaged in the telemedical management of patients with musculoskeletal injuries or polytrauma during acute care and rehabilitation clinics. We established digital ward rounds between an acute care hospital and rehabilitation clinics with live patient presentation. A self-developed questionnaire assessed four domains: overall satisfaction, data security, technical setup, and the rehabilitation process. Each item was rated using a 5-point Likert scale. Participation was voluntary and anonymous.

**Results:**

A total of 39 participants completed the survey, with 23 respondents (59%) from a rehabilitation institute and 14 (41%) from an acute care hospital. The majority were physicians (*n* = 32, 82.1%), with the most common age group being 50–60 years (*n* = 12, 30.8%). Overall satisfaction with the telemedicine process was high, with 31 participants rating their experience as either 4 or 5 (very satisfied). Only one respondent expressed concerns regarding data security, while the majority were neutral or disagreed with statements suggesting data security breaches. The reported preparation time for televisits was generally between 5 and 10 min.

**Conclusion:**

Healthcare professionals reported high satisfaction with telemedicine-supported digital ward rounds and expressed minimal concern regarding data security. Televisits are perceived as a feasible and practical tool for enhancing communication between acute care hospitals and rehabilitation centers, potentially improving the quality and continuity of musculoskeletal and trauma rehabilitation.

**Supplementary Information:**

The online version contains supplementary material available at https://doi.org/10.1007/s00068-026-03084-w.

## Background

The period following hospital discharge represents a critical phase in patient care, particularly in surgical settings. During this time, loss to follow-up remains a significant challenge, often resulting from irregular post-discharge consultations and the lack of direct contact between patients and their surgeons [1–4]. This disruption in continuity of care poses several problems, especially when considering the limited resources available in healthcare systems. Moreover, logistical issues such as time constraints and geographical barriers further complicate effective postoperative monitoring. Telemedicine offers a promising solution to these challenges and has grown substantially in recent years, particularly during the Covid-19 pandemic [5–8]. It also helps to overcome geographic and resource constraints. Driven by technological advancements and proliferation of mobile technology there is increasing need for accessible healthcare solutions [9–12]. By facilitating remote consultations and enhancing communication between patients and their surgeons, telemedicine can reduce the impact of loss to follow-up and ensure more consistent postoperative care even when traditional in-person consultations are difficult. High levels of patient and provider satisfaction have been reported, along with notable reductions in travel time, costs and the frequency of postoperative visits [13–15]. Most existing literature focuses on hospital-to-home transitions, highlighting that adverse events after discharge are common and can result in readmissions, emergency department revisits, patient dissatisfaction, and medication errors [16, 17]. Televisits, and newer solutions such as smartphone applications, can enhance patient safety and the completeness of discharge processes [18–20]. Another significant field of modern telemedicine is telerehabilitation, which has for example a big influence in the recovery of orthopedic patients requiring physical therapy [21–25]. Moreover, telemedicine in general has the potential to bridge not only the gap between surgeons and patients, but also between acute hospitals and rehabilitation clinics, providing a seamless transition of care and improving follow-up processes. This paper aims to explore the potential benefits, challenges, and implications of televisits between acute hospitals and rehabilitation clinics within the broader context of modern healthcare systems [11]. However, conclusive evidence that telerehabilitation is fully comparable to standard face to face treatment remains limited [26].

## Televisits

### Live digital ward rounds

We established weekly live digital ward rounds connecting patients from an acute care hospital with healthcare professionals at affiliated rehabilitation clinics. Each session is scheduled at a fixed time, and while there is one acute care hospital, multiple rehabilitation clinics participate, each with its own designated appointment. Only patients with an upcoming discharge are enrolled in these ward rounds, which are conducted as bedside video calls, linking the patient with staff from both the acute care hospital and the rehabilitation clinic. Televisits are performed on the surgical ward and not during intensive care periods. Digital ward rounds (televisits) were conducted while patients were admitted to the acute care hospital. During these televisits, trauma patients were presented by the acute care team via secure video conferencing to physicians from the collaborating rehabilitation clinics to enable early interdisciplinary discussion and rehabilitation planning.

Following transfer to the rehabilitation clinic, continuity of care was ensured through weekly in-person visits by a trauma surgeon at the rehabilitation center. After discharge from rehabilitation, all patients were routinely followed up in the outpatient clinic of the acute care hospital.

### Participants and roles

At the acute care hospital, the typical team includes the attending ward physician (usually a trauma resident), a trauma surgery specialist, and nursing staff. At the rehabilitation clinic, the team typically consists of the rehabilitation physician, one or two nurses, and a physiotherapist. If needed, additional specialists such as psychiatrists or neurologists may join to address specific aspects of the patient’s rehabilitation. This multidisciplinary approach ensures that all relevant aspects of care are considered.

### Role of family or caregivers

Family members or caregivers are invited to join the teleconsultation, though their participation is optional and not always regular. When present, they provide valuable support for the patient, particularly in discussing rehabilitation goals, patient motivation, and any home care requirements.

### Lack of dedicated liaison and coordination

Currently, there is no dedicated liaison personnel bridging the acute care and rehabilitation teams. Communication between the two services primarily takes place during the digital ward round itself, with each team contributing information and recommendations based on their respective expertise. In the future, a more formalized liaison role—such as a rehabilitation physician or rehab nurse integrated into both teams—could enhance coordination during triage and ensure a more seamless transfer of care from the acute care phase to rehabilitation.

### Process and Preparation

Each consultation lasts approximately ten minutes. Prior to the session, relevant patient information (including medical history, trauma mechanism, performed interventions or surgeries, current treatments, and rehabilitation goals) is provided to the rehabilitation clinic team via email, typically the day before the consultation. This ensures that rehabilitation staff are prepared and can contribute meaningfully to the discussion, despite the lack of a shared electronic health record or imaging system.

### Focus of the consultation

During the televisit, the team discusses the patient’s medical history, trauma mechanism, reason for admission, surgeries or interventions performed, ongoing treatments and medications, rehabilitation plan and schedule, psychological status, and anticipated discharge date. This collaborative approach ensures that the rehabilitation team is fully informed and can adjust the rehabilitation plan as needed, based on the latest clinical information from the acute care team.

### Referral

Referral to inpatient rehabilitation is based on several criteria, including the type and extent of injuries (mono- or polytrauma), functional limitations, need for nursing care, pain, comorbidities, age, cost coverage, rehabilitation potential and goals. The indication is a medical decision made by the treatment team including surgeons, physiotherapists, nursing staff, and social services. Planning for discharge and rehabilitation begins early after admission, once the acute treatment phase is stable. If inpatient rehabilitation is needed, the medical team applies to the relevant insurance provider, a process that usually takes about two days. The interdisciplinary team then discusses the plan with the patient, including the choice of rehabilitation facility. If the insurance does not approve, private payment or a reconsideration appeal can be pursued.

## Methods

This study utilized a cross-sectional survey design, which involves collecting data from participants at a single point in time, to capture the perspectives and experiences of healthcare professionals involved in above mentioned televisits. Data were collected using a specifically developed questionnaire (Supplement).

### Population

The target population were healthcare professionals actively engaged in televisits, including medical doctors, physiotherapists and nurses, who either work in an acute care hospital or a rehabilitation clinic. Involved medical doctors were either residents or specialists from different subspecialties, including trauma surgery, rehabilitation medicine, psychiatry and neurology. All medical professionals working in the acute care hospital or an involved rehabilitation clinic, who were actively involved in televisits in the last year were recruited via email. Only fully completed questionnaires were included in the analysis. Patients were yet not involved in this study. Given that the study targeted a small, clearly defined population, a formal sample size calculation was not considered necessary, as all eligible participants were invited to participate.

### Questionnaire

The questionnaire was specifically developed for this study to assess healthcare professionals’ perspectives on telemedicine-supported digital ward rounds in trauma care. It comprised 37 questions and was created by our study group with input from physicians, nurses, and rehabilitation professionals to ensure that all relevant aspects of the televisit process were addressed. It included a mix of both quantitative (Likert scale and multiple-choice) and qualitative (open-ended) questions to capture participants’ satisfaction, technical concerns, and suggestions for improvement. The aim was to assess four key domains: technical setup, satisfaction with the televisit process, the impact on rehabilitation, and the practical aspects of conducting televisits. A pilot phase was conducted to ensure clarity, and minor adjustments were made based on initial feedback from participants. The final version was translated into English for broader accessibility and use in the study.

### Data collection

The questionnaire was distributed electronically via Typeform to healthcare professionals involved in the telemedicine-supported digital ward rounds. Data collection occurred over a period from September 17, 2024, to October 9, 2024. Participation in the survey was voluntary, and all responses were anonymous to ensure confidentiality and minimize potential bias. The questionnaire was sent to all eligible participants via email, with one reminder sent midway through the data collection period to maximize response rates.

### Data analysis

Quantitative data were analyzed using descriptive statistical methods, including the calculation of means, standard deviations, and frequencies for the Likert-scale and closed-ended questions. This allowed us to summarize the general trends in participant responses.

For the qualitative responses, a thematic analysis was conducted. This involved identifying, analyzing, and reporting patterns or themes within the open-ended responses. Thematic analysis was used to gain a deeper understanding of participants’ subjective experiences and to provide more context to the quantitative findings. Given the primarily exploratory nature, descriptive design and the limited sample size, neither inferential or subgroup analyses were conducted. All data analysis was performed using R software (version 3.6), ensuring a rigorous and transparent approach to both the quantitative and qualitative analysis.

## Results

**Fig. 1:**
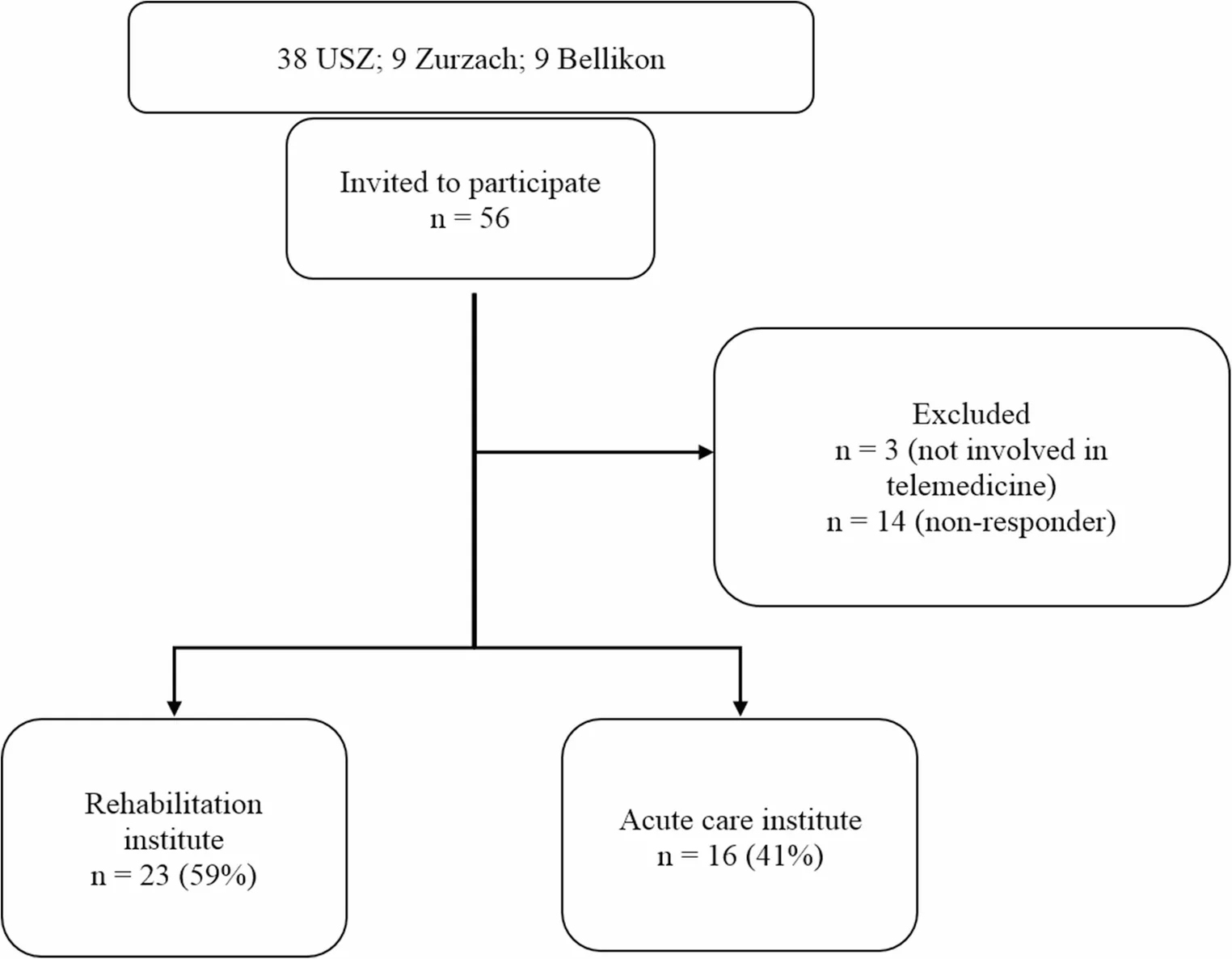
Flow chart of invited healthcare professionals and completed responses

The questionnaire was distributed to 56 eligible participants, of whom three were excluded due to non-involvement in telemedical ward rounds. A total of 39 responses were included in the analysis, yielding a response rate of 74% (Fig. 1). Among respondents, 16 (41%) worked in acute care hospitals and 23 (59%) in rehabilitation clinics. The majority were medical doctors (*n* = 32, 82%), with a nearly balanced gender distribution (20 males [51%], 19 females [49%]). The most common age groups were 20–30 and 50–60 years. The mean duration of participation in tele-rounds was 3.7 years. At the time of the survey, 90% of participants were employed full-time. Detailed demo-graphic information is presented in Table 1.

**Table 1:** Demographic data of involved healthcare professionals

n	39
Acute care hospital, n (%)	16 (41)
Rehabilitation institute, n (%)	23 (59)
Medical doctor, n (%)	32 (82.1)
Nurse, n (%)	7 (17.9)
Female sex, n (%)	19 (48.7)
Age [years], n(%)	
20-30	10 (25.6)
30-40	9 (23.1)
40-50	4 (10.3)
50-60	12 (30.8)
above 60	4 (10.3)
Experience with telemedicine in years, mean (SD)	3.69 (2.27)
Full-time employment, n (%)	35 (89.7)

The survey on televisits comprising healthcare professionals revealed generally high satisfaction and feasibility of telemedical ward rounds between acute care and rehabilitation clinics.

Overall satisfaction with televisits was high, with a mean score of 4.2 out of 5, and the majority of respondents rated their experience as very satisfied (4 or 5, Table 2).

**Table 2:** Summary of televisit survey results showing satisfaction, technical quality, clinical impact, and acceptance among healthcare professionals

Aspect	Mean/Percentage	Additional Information
Satisfaction with Televisits	4.2/5	Majority rated 4 or 5 (very satisfied)
Satisfaction with Technical Setup	4.3/5	High satisfaction with setup
Frequency of Technical Problems	15% “Never”	Majority reported no issues
Required Preparation Time/Patient	5–10 min	Most respondents (60%) rated this time
Meaningful Appointment	3.6/5	Moderate agreement on clinical meaningfulness
Improved Follw-up Treatment	4.2/5	Significant improvement documented
Promotes Interdisciplinary Collaboration	3.9/5	Supports teamwork and communication
Data Security Concerns	9%	Few participants expressed privacy concerns
Would Recommend Concept	87% yes	High endorsement for continued use
Plan to Continue Using	91% yes	Strong commitment for future telemedicine use

**Fig. 2:**
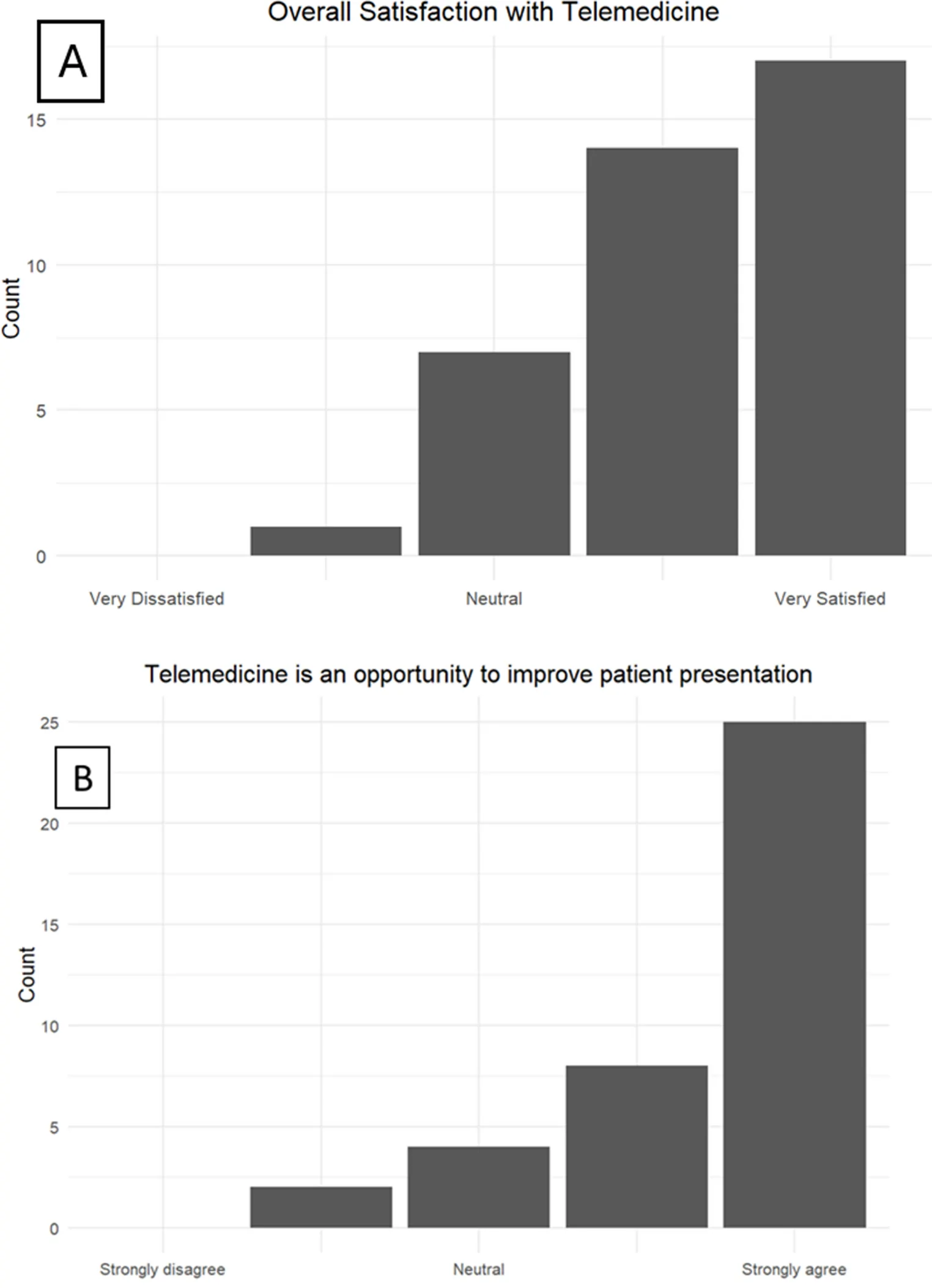
**A**: Most participants are satisfied and very satisfied in general with telemedicine, **B**: Most participants strongly agree that telemedicine serves as an opportunity to improve patient presentation and discussion

Lower satisfaction was primarily associated with bedside limitations—such as limited direct patient interaction—or insufficient focus on medical issues. Most participants rated tele-rounds as a meaningful form of clinical interaction (mean score: 4.1, Table 2). Furthermore, they were perceived to enhance the quality of patient presentation (mean score: 4.4, Fig. 2B).

In the context of the digital ward rounds, patient presentation refers to the process where the healthcare team reviews and discusses the patient’s medical history, trauma mechanism, performed interventions or surgeries, ongoing treatments, rehabilitation plans, and other relevant clinical details. This is done in a collaborative format where both the acute care hospital team and the rehabilitation clinic team contribute their expertise. The goal is to ensure that all relevant information is shared for the benefit of the patient’s ongoing care and rehabilitation.

Satisfaction with the technical setup was similarly solid at 4.3 out of 5, reflecting confidence in the system infrastructure. Technical problems were infrequent, with 15% reporting no issues at all, indicating overall system stability and reliability (Table 2).

The preparation time required per patient was typically 5 to 10 min, as reported by approximately 60% of participants, which was deemed appropriate for workflow integration (Table 2).

Preparation time per patient refers to the time required by healthcare professionals to gather and review the necessary information before participating in the digital ward round. This includes reviewing the patient’s medical history, understanding the rehabilitation needs, and preparing relevant documentation (such as patient records and imaging). The questionnaire asked participants to estimate how much time they typically spend preparing for each televisit, with responses indicating that preparation usually takes between 5 and 10 min.

Clinically, the televisits were considered meaningful, with a moderate agreement score of 3.6 out of 5 regarding their value as a clinical appointment (Table 2). Notably, telemedical ward rounds significantly improved follow-up treatment coordination, scoring 4.2 on average (Table 2). Interdisciplinary collaboration was also enhanced, with a mean score of 3.9 out of 5, underscoring the method’s potential to strengthen team communication (Table 2).

Concerns regarding data security and privacy were low, with only 9% expressing apprehension, indicating acceptable trust in the digital platform (Table 2). Importantly, acceptance of the tele-round concept was high, with 87% of respondents willing to recommend its use and 91% planning to continue utilizing the system, demonstrating strong sustainability and user endorsement (Table 2).

Participants provided the following reasons for not recommending televisits: time-consuming, limited impact and discrimination against more complex or high-maintenance patients. The requested improvements included the pre-transmission of images and medical data, as well as a bilateral exchange, including also the presentation of patients in rehabilitation clinics for follow-ups.

The majority of respondents reported televisits should encompass the following information: age, sex, profession, trauma mechanism/reason for admission, diagnosis/injuries, admission day, performed interventions/surgeries, past medical history, complications, current medication, current limitations, planned date of discharge, caregiver, rehabilitation potential, motivation, malcompliance, rehabilitation schedule, pain situation, next follow-ups.

These results highlight the practical benefits and broad acceptance of telemedical ward rounds in facilitating effective and efficient communication across care settings while maintaining clinical quality and workflow efficiency.

## Discussion

The high satisfaction rates reported in this study highlight the growing acceptance of televisits among healthcare professionals. With an average satisfaction score of 4.2 out of 5, most participants considered televisits as a reasonable alternative to in-person appointments and a valuable opportunity to enhance patient presentation. This aligns with previous studies emphasizing the effectiveness of telemedicine in improving patient engagement and satisfaction while reducing logistical challenges [27].

Technical reliability was generally rated positively, with few participants reporting technical issues. This supports the notion that technological advancements have made telemedicine increasingly feasible and reliable for routine clinical use [28]. While some concerns about data security remain, most participants expressed little to no worries about privacy issues, which aligns with findings from other studies suggesting that well-designed platforms can ensure data protection [29, 30].

The perceived benefits of televisits, such as reducing the length of stay and enhancing interdisciplinary collaboration, are consistent with existing literature advocating for telemedicine’s role in streamlining the continuum of care from acute hospitals to rehabilitation clinics. In many clinical settings, limited space, ongoing procedures, or infection control measures can restrict healthcare professionals’ bedside access to patients. Further, remote consultations are always limited by the absence of physical contact. By identifying these barriers and adapting workflows, such as scheduling visits during less busy periods or using portable telemedicine equipment, bedside access challenges could be mitigated, ensuring that remote consultations do not compromise patient care, safety or staff efficiency. However, a more integrated platform for data sharing (e.g. shared electronic health record), and better inclusion of patients’ families should be improved in the future. Overall, the findings of this study support the continued use and development of televisits, particularly in enhancing interdisciplinary collaboration and improving the overall rehabilitation process. Ensuring technological stability, privacy, and patient-centered care will be essential in maximizing the potential of telemedicine.

### Limitations

This study has several limitations that should be acknowledged. The use of a self-developed survey limits the external validity, and formal instrument validation was limited by sample size and feasibility considerations. The absence of inferential statistics and subgroup analysis restricts the depth of conclusions that can be drawn. Moreover, the study focused on healthcare professional perspectives without direct patient input, which may affect generalizability.

Challenges related to bedside access and technical barriers identified in the study remain to be addressed through workflow adaptations and further investigation. Future research should incorporate larger, more diverse populations, rigorous instrument validation, and quantitative analyses to substantiate and extend these initial findings.

## Conclusion

This study highlights the potential of telemedical ward rounds in improving communication and continuity of care between acute care hospitals and rehabilitation clinics. Healthcare professionals reported high satisfaction with the process, particularly in terms of patient presentation, interdisciplinary collaboration, and overall efficiency. Despite minor technical issues, concerns about data security were minimal, and the time investment was generally deemed appropriate. While the findings suggest that telemedicine could reduce delay in transfer from acute care to the rehabilitation clinic and might be associated with improved rehabilitation outcomes, these conclusions are based on healthcare professionals’ perceptions and a self-developed survey. Further research, including patient involvement and larger sample sizes, is needed to validate these results and assess the broader impact of televisits on patient care.

## Supplementary Information

Below is the link to the electronic supplementary material.

Supplementary Material 1

## Data Availability

No datasets were generated or analysed during the current study.

## References

[1] Aaland MO, Marose K, Zhu TH. The lost to trauma patient follow-up: a system or patient problem. J Trauma Acute Care Surg. 2012;73(6):1507–11. 10.1097/TA.0b013e31826fc928. 23147179

[2] Smith SM, Zhao X, Kenzik K, Michael C, Jenkins K, Sanchez SE. Risk factors for loss to follow-up after traumatic injury: an updated view of a chronic problem. Surgery. 2024;175(5):1445–53. 10.1016/j.surg.2024.01.034 PMC11533560 38448279

[3] Reed M, Huang J, Brand R, Graetz I, Jaffe MG, Ballard D et al. Inpatient-outpatient shared electronic health records: telemedicine and laboratory follow-up after hospital discharge. Am J Manag Care [Internet]. 2020 [cited 2025 Oct 8];26(10):E327–32. Available from: https://pubmed.ncbi.nlm.nih.gov/33094945/10.37765/ajmc.2020.88506 PMC11594565 33094945

[4] Cam H, Kempen TGH, Eriksson H, Abdulreda K, Franzon K, Gillespie U. Assessment of requests for medication-related follow-up after hospital discharge, and the relation to unplanned hospital revisits, in older patients: a multicentre retrospective chart review. BMC Geriatr [Internet]. 2021 Dec 1 [cited 2025 Oct 8];21(1). Available from: https://pubmed.ncbi.nlm.nih.gov/34724895/10.1186/s12877-021-02564-5 PMC8561898 34724895

[5] Glasgow S, Saha P, Moore H, James B, Newsholme W, Zayed H. Perception and acceptability of virtual ward round reviews of vascular inpatients to preserve resources and reduce infection risk in the COVID-19 era. Eur J Vasc Endovasc Surg. 2021. 10.1016/j.ejvs.2020.11.014. PMC7666534 33243597

[6] Bezerra GMF, De Lucena Feitosa ES, Vale Catunda JG, Nogueira Sales Graça C, De Lucena P, Bezerra Neto AG et al. Telemedicine Application and Assessment During the COVID-19 Pandemic. Stud Health Technol Inform [Internet]. 2022 Jun 6 [cited 2025 Oct 8];290:854–7. Available from: https://pubmed.ncbi.nlm.nih.gov/35673139/10.3233/SHTI220200 35673139

[7] Brunet F, Malas K, Desrosiers ME. Will telemedicine survive after COVID-19? Healthc Manag forum [Internet]. 2021 Sep 1 [cited 2025 Oct 8];34(5):256–9. Available from: https://pubmed.ncbi.nlm.nih.gov/34340570/10.1177/08404704211031264 34340570

[8] Gareev I, Gallyametdinov A, Beylerli O, Valitov E, Alyshov A, Pavlov V et al. The opportunities and challenges of telemedicine during COVID-19 pandemic. Front Biosci (Elite Ed) [Internet]. 2021 Dec 1 [cited 2025 Oct 8];13(2):291–8. Available from: https://pubmed.ncbi.nlm.nih.gov/34937315/10.52586/E885 34937315

[9] Yau T, Chan J, McIntyre M, Bhogal D, Andreoli A, Leochico CFD, et al. Adverse events associated with the delivery of telerehabilitation across rehabilitation populations: a scoping review. PLoS One. 2024;19(11):e0313440. 10.1371/journal.pone.0313440 PMC11575805 39561143

[10] Mahar JH, Rosencrance GJ, Rasmussen PA. Telemedicine: past, present, and future. Cleve Clin J Med. 2018. 10.3949/ccjm.85a.17062. 30526749

[11] Stoltzfus M, Kaur A, Chawla A, Gupta V, Anamika FNU, Jain R. The role of telemedicine in healthcare: an overview and update. Egypt J Intern Med. 2023;35:49. 10.1186/s43162-023-00234-z.

[12] Shaver J. The state of telehealth before and after the COVID-19 pandemic. Primary Care - Clinics in Office Practice. 2022. 10.1016/j.pop.2022.04.002. PMC9035352 36357058

[13] Sharareh B, Schwarzkopf R. Effectiveness of telemedical applications in postoperative follow-up after total joint arthroplasty. J Arthroplasty. 2014;29(5):918–22.e1. 10.1016/j.arth.2013.09.019. 24342278

[14] Gunter RL, Chouinard S, Fernandes-Taylor S, Wiseman JT, Clarkson S, Bennett K, et al. Current use of telemedicine for Post-Discharge surgical care: A systematic review. J Am Coll Surg. 2016;222(5):915–27. 10.1016/j.jamcollsurg.2016.01.062 PMC5660861 27016900

[15] Sathiyakumar V, Apfeld JC, Obremskey WT, Thakore RV, Sethi MK. Prospective randomized controlled trial using telemedicine for follow-ups in an orthopedic trauma population: a pilot study. J Orthop Trauma. 2015;29(3):e139–e45. 10.1097/BOT.0000000000000189. 24983434

[16] Forster AJ, Murff HJ, Peterson JF, Gandhi TK, Bates DW. The incidence and severity of adverse events affecting patients after discharge from the hospital. Ann Intern Med. 2003;138:161–7. 10.7326/0003-4819-138-3-200302040-00007. 12558354

[17] Alqenae FA, Steinke D, Keers RN. Prevalence and nature of medication errors and medication-related harm following discharge from hospital to community settings: a systematic review. Drug Saf. 2020. 10.1007/s40264-020-00918-3. PMC7235049 32125666

[18] Bhatia K, Nawaz K, Waldman LM, Khandakar S, Sitt H, Desai N, et al. A quality improvement project to improve hospital-to-home transitions using discharge televisits. Hosp Pediatr. 2023;13(8):724–32. 10.1542/hpeds.2022-006860. 37435664

[19] Baniasadi T, Hassaniazad M, Rostam Niakan Kalhori S, Shahi M, Ghazisaeedi M. Developing a mobile health application for wound telemonitoring: a pilot study on abdominal surgeries post-discharge care. BMC Med Inform Decis Mak. 2023;23:103. 10.1186/s12911-023-02199-z. PMC10237057 37268995

[20] Heath J, Dancel R, Stephens JR. Postdischarge phone calls after pediatric hospitalization: an observational study. Hosp Pediatr. 2015;5(5):241–8. 10.1542/hpeds.2014-0069. 25934807

[21] Seron P, Oliveros MJ, Gutierrez-Arias R, Fuentes-Aspe R, Torres-Castro RC, Merino-Osorio C, et al. Effectiveness of telerehabilitation in physical therapy: a rapid overview. Phys Ther. 2021;101(6):pzab053. 10.1093/ptj/pzab053. PMC7928601 33561280

[22] Chughtai M, Kelly JJ, Newman JM, Sultan AA, Khlopas A, Sodhi N, et al. The role of virtual rehabilitation in total and unicompartmental knee arthroplasty. J Knee Surg. 2019;32(01):105–10. 10.1055/s-0038-1637018. 29548061

[23] Ouendi N, Avril E, Dervaux B, Pudlo P, Wallard L. Effectiveness of Telerehabilitation Programs in Elderly with Hip or Knee Arthroplasty: A Systematic Review. Telemed J E Health [Internet]. 2024 Jun 1 [cited 2025 Oct 8];30(6):1507–21. Available from: https://pubmed.ncbi.nlm.nih.gov/38574249/10.1089/tmj.2023.0622 38574249

[24] De Berardinis L, Senarighi M, Ciccullo C, Forte F, Spezia M, Gigante AP. Fast-track surgery and telerehabilitation protocol in unicompartmental knee arthroplasty leads to superior outcomes when compared with the standard protocol: a propensity-matched pilot study. Knee Surg Relat Res [Internet]. 2022 Dec 1 [cited 2025 Oct 8];34(1). Available from: https://pubmed.ncbi.nlm.nih.gov/36510279/10.1186/s43019-022-00173-z PMC9743114 36510279

[25] Tsuge T, Yamamoto N, Taito S, Miura T, Shiratsuchi D, Yorifuji T. Efficacy of telerehabilitation for patients after hip fracture surgery: A systematic review and meta-analysis. J Telemed Telecare [Internet]. 2025 Mar 1 [cited 2025 Oct 8];31(2):174–83. Available from: https://pubmed.ncbi.nlm.nih.gov/37416946/10.1177/1357633X231181632 37416946

[26] Morri M, Ruisi R, Culcasi A, Peccerillo V. The Effectiveness of Telerehabilitation for Functional Recovery After Orthopedic Surgery: A Systematic Review and Meta-Analysis. Telemed reports [Internet]. 2024 Mar 1 [cited 2025 Oct 8];5(1):78–88. Available from: https://pubmed.ncbi.nlm.nih.gov/38558953/10.1089/tmr.2023.0057 PMC10979691 38558953

[27] Dorsey ER, Topol EJ. Telemedicine 2020 and the next decade. Lancet. 2020;395(10227):859. 10.1016/S0140-6736(20)30424-4. 32171399

[28] Smith AC, Thomas E, Snoswell CL, Haydon H, Mehrotra A, Clemensen J, et al. Telehealth for global emergencies: implications for coronavirus disease 2019 (COVID-19). J Telemed Telecare. 2020;26(5):309–13. 10.1177/1357633X20916567. PMC7140977 32196391

[29] Ahmad A, Mustafa A, Qureshi AA, Naseem A, Rana AR, Akhtar M, et al. Telemedicine practice: current challenges of consent and Autonomy, patient privacy and data security worldwide. J Soc Prev Advocacy Res KEMU. 2024;3(1):47–53.

[30] Andreadis K, Muellers KA, Lin JJ, Mkuu R, Horowitz CR, Kaushal R, et al. Navigating privacy and security in telemedicine for primary care. Am J Manag Care. 2024;30:SP459–63. 6 Spec No.). 10.37765/ajmc.2024.89553 38820187

