# Supplementary material for: From surgery to rehabilitation: a cross-sectional survey on the feasibility of live digital ward rounds between acute care and rehabilitation clinics in trauma patients
Source: Eur J Trauma Emerg Surg. 2026 Mar 17;52(1):100. doi: 10.1007/s00068-026-03084-w (PMC13675343; doi:10.1007/s00068-026-03084-w)
Supplement: Supplementary file 1 [file 68_2026_3084_MOESM1_ESM.pdf]

Questionnaire

1. Work place
    - Acute care institue
    - Rehabilitation institute
    - Other

2. Job/ Profession
    - Medical Doctor
    - Physiotherapist
    - Nurse
    - Other

3. Other Job/ Profession
    - ________________

4. Gender
    - Male
    - Female
    - Divers

5. Age in years
    - 20-30
    - 30-40
    - 40-50
    - 50-60
    - Above 60

6. Participation in telemedicine since
    - 0 to 10 years

7. Employment type
    - Full time
    - Part Time

8. Satisfaction with technical setup
    - 1 to 5 stars

9. Audio quality
    - 1 to 5 stars

10. Image quality
    - 1 to 5 stars

11. Occurance of technical problems
    - never
    - seldom
    - sometimes
    - often
    - always

12. Problems with technical setup
    - __________________

13. I have concerns regarding data security
    - Strongly disagree
    - disagree
    - neutral
    - agree
    - strongly agree

14. Telemedicine can replace personal visits
    - Strongly disagree
    - disagree
    - neutral
    - agree
    - strongly agree

15. Other participants are not on time
    - never
    - seldom
    - sometimes
    - often
    - always

16. Cost settlement
    - We can bill telemedicine
    - We would like to bill telemedicine
    - We don't bill and we we're not interested in
    - Other

17. Documentation
    - Saving video files
    - Written documentation
    - other
    - none
    - not sure

18. Preparation time per patient in minutes
    - Under 5
    - 5 to 10
    - 10 to 15
    - 15 to 20
    - More than 20

19. Overall satisfaction with telemedicine
    - 1 to 5 stars

20. What could be better or improved
    - _______________________

21. Telemedicine is a meaningful appointment
- Strongly disagree
- disagree
- neutral
- agree
- strongly agree

22. Telemedicine is a good opportunity to present patients
- Strongly disagree
- disagree
- neutral
- agree
- strongly agree

23. Because of telemedicine I feel better prepared for my patients
- Strongly disagree
- disagree
- neutral
- agree
- strongly agree

24. Because of telemedicine I have an overall time reduction
- Strongly disagree
- disagree
- neutral
- agree
- strongly agree

25. Because of telemedicine I have less follow-up questions
- Strongly disagree
- disagree
- neutral
- agree
- strongly agree

26. The time frame of telemedicine is
    - too short
    - good
    - too long

27. Telemedicine improves the aftercare/ rehabilitation process/ further treatment
- Strongly disagree
- disagree
- neutral
- agree
- strongly agree

28. Because of telemedicine, triage in rehabilitation institues is better
- Strongly disagree
- disagree
- neutral
- agree
- strongly agree
-

29. Because of telemedicine, patients get faster access to a free rehabilitation place
- Strongly disagree
- disagree
- neutral
- agree
- strongly agree

30. Telemedicine improve interdisciplinary and interprofessionell work and cooperation
- Strongly disagree
- disagree
- neutral
- agree
- strongly agree

31. What are the keypoints that should be mentioned in a telemedicine consultation

    ____

32. Would you recommend the concept telemedicine
    - no
    - rather no
    - Half/half
    - rather yes
    - yes

33. Why would you not recommend the telemedicine concept?

    ____

34. Do you want to continue your participation in telemedicine
    - yes
    - neutral
    - no

35. Why don’t you want to continue your participation?

    ____

36. A transfer of documents would help me
    - before
    - live/ realtime
    - after
    - neutral
    - no

37. Other

    ____
